# A Clinicopathological Study with Risk-Stratified Staging of Pediatric Hepatoblastoma: A 5-Year Experience from a Tertiary Cancer Center

**DOI:** 10.30699/IJP.2023.1972340.3005

**Published:** 2023-06-20

**Authors:** Geeta V Patil Okaly, Akina Prakash, C Akshatha, Ashwini Nargund, Libin Babu Cherian, S Balu, AR Arun Kumar

**Affiliations:** 1 *Department of Pathology, Kidwai Memorial Institute of Oncology, Rajiv Gandhi University of Health Sciences, Bangalore, Karnataka, India*; 2 *Department of Pediatric Oncology, Kidwai Memorial Institute of Oncology, Rajiv Gandhi University of Health Sciences, Bangalore, Karnataka, India*

**Keywords:** Fetal, Hepatoblastoma, Macrotrabacular, Teratoid

## Abstract

**Background & Objective:**

Hepatoblastoma encompasses 1% of pediatric malignancies and is the most common liver malignancy in children. Ninety percent of cases are younger than 5 years of age. Clinical and pathological risk stratification forms a crucial role in determining the treatment strategy. This study aimed to assess the clinicopathological profile of hepatoblastoma with risk stratification and follow-up in children.

**Methods:**

A retrospective evaluation was performed on all pediatric patients diagnosed as hepatoblastoma between 2016 and 2020 in our institution. Clinical, radiological, biochemical, pathological, and treatment data were analyzed. Cases were stratified based on the SIOPEL protocol and compared with the outcome.

**Results:**

The median age of all children was 1 year, the male-to-female ratio was 2.3:1, and elevated α-fetoprotein (AFP) was observed in all cases. SIOPEL risk stratification showed that 50% of children were at high risk. The histopathological types were fetal (30%), embryonal (20%), and macrotrabecular (5%) patterns under epithelial type and mixed epithelial and mesenchymal type (45%) with 1 case showing teratoid features. During the follow-up period, 6 out of the 7 children who died, belonged to the high-risk SIOPEL category, and 5 presented a mixed epithelial and mesenchymal pattern.

**Conclusion:**

Our study found a significant correlation between clinicopathological data, histopathological patterns, and outcomes. Accordingly, histopathological patterns could be considered one of the criteria for risk stratification. Histopathological risk stratification indicators (such as SIOPEL and PRETEXT) have strong prognostic and predictive outcomes; hence, our study emphasizes such parameters to aid oncologists

## Introduction

Hepatoblastoma accounts for 1% of all pediatric malignancies, frequently manifests in patients less than 5 years of age, and is, to some extent, more common in males than in females (1,2). Survival rates have increased to 80% from 20% over the past few years; hence, the management of tumors has been an area of prospective research interest in pediatric oncology. Due to an expansive multifaceted and integrated effort spanning several years in various centers, it has become feasible to obtain improved resection rates where a total exenteration of this liver tumor is essential for treatment.

As the incidence of hepatoblastoma is very low, population-based epidemiological studies are difficult to perform; hence, researchers have not been able to find the exact etiology. Some of the risk aspects comprise low birth weight, prematurity, maternal smoking, alcohol consumption, and use of oral contraceptives (3,4). It is also accompanied by some syndromes, including familial adenomatous polyposis syndromes, TP53 mutation syndrome (Li-Fraumeni syndrome), Wilms tumor-associated syndromes (such as Beckwith-Wiedemann syndrome and Edward syndrome). Exceptional cases of sporadically occurring hepatoblastoma show links with oral contraceptive use through pregnancy, fetal alcohol syndrome, hormone treatment for impotence, and maternal liver transplantation coalesced with immunosuppressive management during pregnancy (5). 

The SIOPEL (International Childhood Liver Tumors Strategy Group) introduced the PRETEXT (pretreatment extent of disease) staging system, which is extensively used for risk stratification and treatment of hepatoblastoma (6). The PRETEXT system is constructed on computed tomography (CT) scans and/or magnetic resonance imaging (MRI; prepared before treatment) and considers the site and dimension of the tumor, vascular invasion, and distant spread as adjudged on imaging. The preparation identifies 4 PRETEXT stages (I-IV), exemplifying the number of sections of the liver that is elaborated by the tumor and depicts the magnitude of the disease in the liver using the following letters: “V” if the tumor extends into the vena cava and/or all 3 hepatic veins, “P” if the main and/or both left and right branches of the portal vein are complicated by the tumor, “C” if there is an involvement of the caudate lobe, “E” if there is an indication of extrahepatic intraabdominal disease, and “M” if there are distant metastases (7,8). The Children's Oncology Group (COG) defined histology as a risk stratification parameter (9). 

In 2017, the Children’s Hepatic Tumors International Collaboration (CHIC) established an amalgamated methodology to stratify patients (5). All these conventions have helped in guiding the oncologist to tailor treatment for each patient and brought in contemporary enhancements in treatment, such as neoadjuvant chemotherapy followed by surgical resection and liver transplantation (9,10). Chemotherapy may diminish tumor bulk, making the tumor resectable, and may lead to the complete disappearance of lung metastases. The tumor response rate to the contemporaneous cisplatin-containing chemotherapy schedules varies from 70% to 90%, conferring to the different series (11-14). Neoadjuvant chemotherapy not only makes the tumor smaller, as well as is subsequently more expected to be completely resected, but also more solid, less disposed to bleeding, and more delineated from the remaining healthy liver parenchyma (15-17). Also, possibly prevailing micrometastases in the lungs are treated promptly. Because of these disagreements, some clinicians currently endorse commencing preoperative chemotherapy after biopsy and conceding decisive surgery after 2-3 months of therapy. This treatment plan was implemented by the SIOPEL. Compared to the SIOPEL methodology, the North American Study Groups still acclaim primary surgery as the initial treatment of hepatoblastoma (18-20).

## Material and Methods

**Fig. 1:**
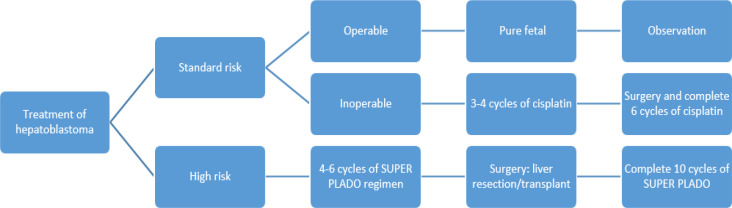
Treatment of hepatoblastoma base on risk parameters

A retrospective analysis was carried out on all children with hepatoblastoma for a period of 5 years from 2016 to 2020 in our institution. Demographic data, α-fetoprotein (AFP) level, CT scan findings, gross features, and histopathological data were collected for all cases and analyzed. Patients were stratified based on the SIOPEL risk stratification protocol. Follow-up data on survival and response to therapy(cisplatin based therapy/ PLADO regimen) were gathered and compared to the clinicopathological aspects (Figure 1).

## Results

The average age of the 20 children was 1 year, and there was a male preference with a male-to-female ratio of 2.3:1. Serum AFP levels were elevated in all the cases. Three children had AFP levels of less than 100 ng/mL, and on follow-up, they died within 1 year of diagnosis. PRETEXT staging was performed using CT scans. Forty-five percent of children presented with PRETEXT III, followed by 40% with PRETEXT IV, 10% with PRETEXT II, and 5% with PRETEXT I.

According to the histopathology results, of the 8 cases presented with PRETEXT IV, 6 had a mixed epithelial and mesenchymal pattern and 2 showed a pure fetal pattern. On follow-up, 4 of these children died within 1 year of diagnosis, 1 presented with recurrence, 2 were lost to follow-up, and 1 child is doing well (Table 1).

According to the histopathology results, 6 out of the 8 cases who presented with PRETEXT IV, showed a mixed epithelial and mesenchymal pattern and 2 demonstrated a pure fetal pattern. On follow-up, 4 of these children died within 1 year of diagnosis, 1 presented with recurrence, 2 were lost to follow-up, and 1 child is doing well (Table 1).

**Fig. 2:**
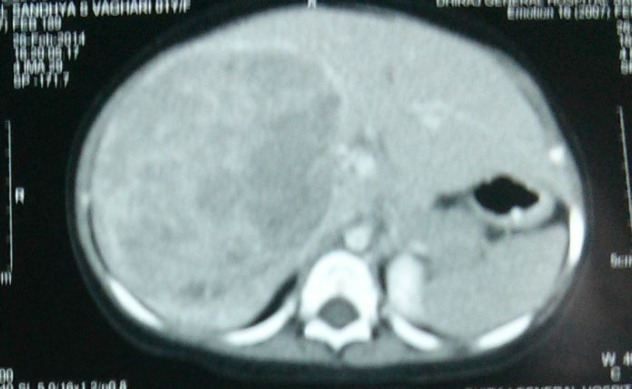
The abdominal CT image showing a heterogeneous enhancing mass in the right lobe of the liver

The average age of the 20 children was 1 year, and there was a male preference with a male-to-female ratio of 2.3:1. Serum AFP levels were elevated in all cases (Tables 2 and 3). Three children had AFP levels of less than 100 ng/mL, and on follow-up, they died within 1 year of diagnosis. PRETEXT staging was performed using CT scans (Figure 2). Forty-five percent of children presented with PRETEXT III, followed by 40% with PRETEXT IV, 10% with PRETEXT II, and 5% with PRETEXT I. 

**Fig. 3:**
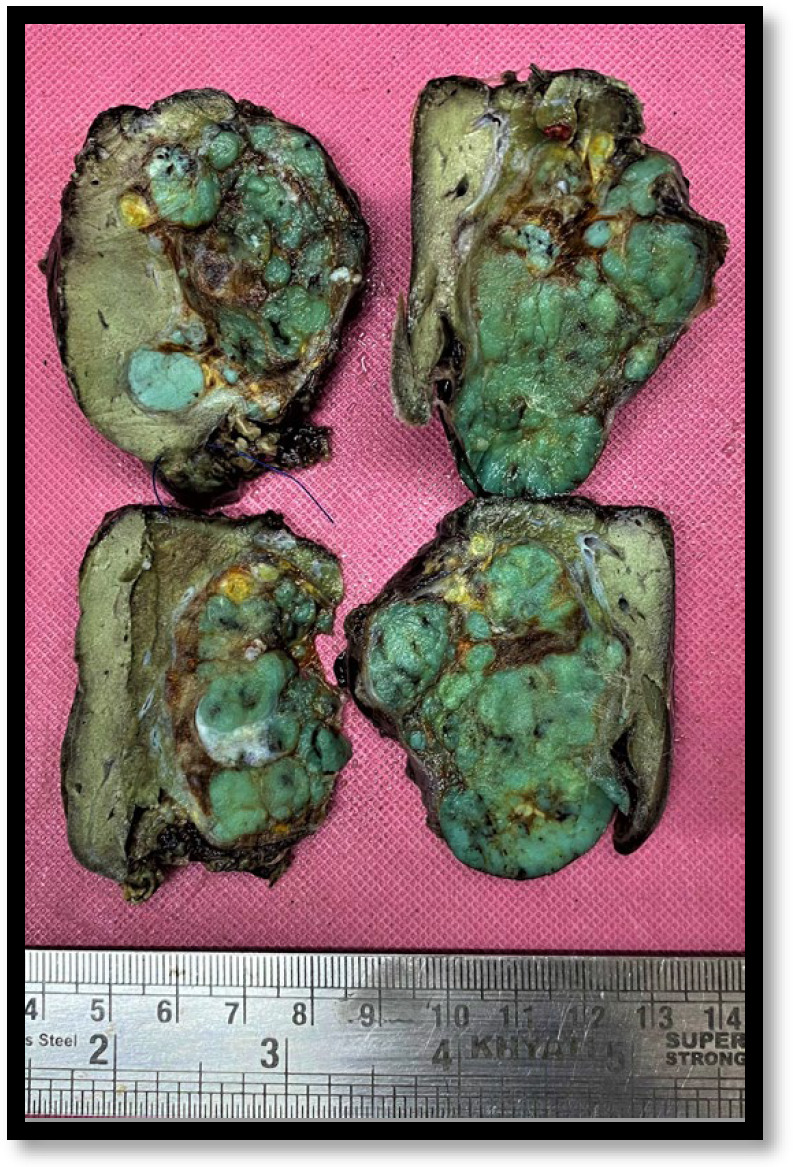
Gross image: The cut surface with a variegated lesion having a nodular appearance with necrosis and hemorrhage. A peripheral rim of normal liver parenchyma seen

Grossly the tumors exhibit a variegated appearance composed of varying sized grey white heterogenous nodules with necrosis and hemorrhage. A peripheral rim of normal liver parenchyma is mostly seen (Figure 3).

**Fig. 4:**
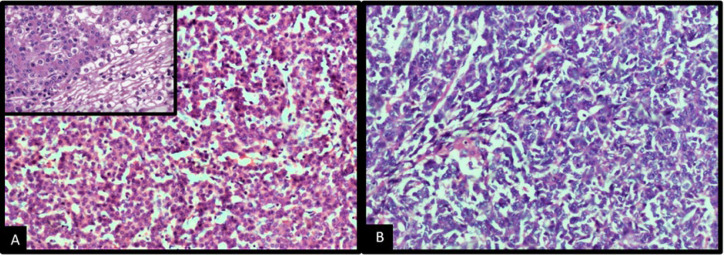
(A) Epithelial type; pure fetal. (B) Epithelial type; fetal and embryonal (H&E; ×40)

**Fig. 5:**
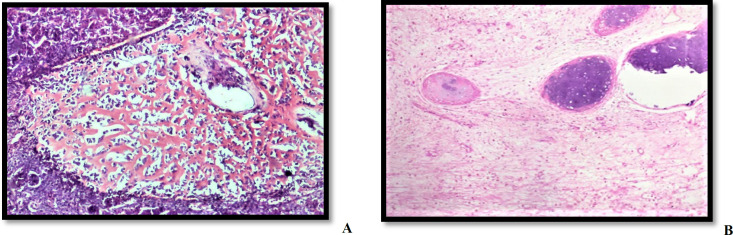
Mixed epithelial and mesenchymal type with (A) osteoid and (B) cartilage (H&E; ×40)

**Fig. 6:**
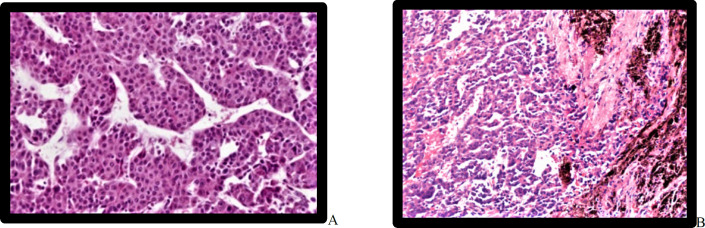
(A) The focal macrotrabecular pattern. (B) Teratoid

The histopathological types according to WHO classification observed 9 cases of mixed epithelial and mesenchymal pattern and 11 cases of epithelial pattern. Patterns observed under epithelial type were 6 pure fetal types (Figure 4A) and 4 mixed fetal and embryonal type (Figure 4B). Of the 9 cases exhibiting a mixed epithelial and mesenchymal pattern, 5 died and 4 are doing well on follow-up. The mesenchymal components included bone, osteoid (Figure 5A), fibrous tissue, and cartilage (Figure 5B). Few cases exhibited post-chemotherapeutic changes, including necrosis, inflammation, and calcification. One case showed fetal with focal macrotrabacular pattern (Figure 6A) and one case exhibited teratoid features (Figure 6B).

**Table 1:** The correlation of follow-up with histopathological patterns and SIOPEL risk categories

Follow-up (20)	Epithelial (11)	Mixed epithelial and mesenchymal (9)	Standard risk (10)	High-risk (10)
Doing good	6	4	9	1
Lost to follow-up	2	Nil	Nil	2
Death	2	5	1	6
Recurrence	1	Nil	Nil	1

**Table 2:** The comparison of the present study with the study done by Hsu *et al.* (30)

	Hsu *et al.* (30) (2020), 45 cases (1998-2018)	The present study, 20 cases (2016-2020)
Median age Male-to-female ratio	1.2 years 1.4:1	1 year 2.3:1
Elevated serum AFP	95% cases	100% cases 3 cases <100 ng/mL
PRETEXT staging frequency	I (4%), II (16%), III (42%), and IV (38%)	I (5%), II (10%), III (45%), and IV (40%)
SIOPEL risk stratification	Standard risk = 25 High-risk = 20	Standard risk = 10 High-risk = 10
Histology types	Epithelial = 75%, Mixed epithelial and mesenchymal = 25%	Epithelial = 55% Mixed epithelial and mesenchymal = 45%
Follow-up	Death = 37% Doing well = 58% Recurrence = 5%	Death = 35% Doing well = 35% Lost to follow-up = 25% Recurrence = 5%

*Note.* AFP = α-fetoprotein; SIOPEL = International Childhood Liver Tumors Strategy Group.

**Table 3:** The comparison of the present study with the study done by Archana *et al.* (31)

Features	Archana *et al.* (31), 10 case series	The present study
Median age Male-to-female ratio	11 months 2.3:1	1 year 2.3:1
Elevated serum AFP	90% cases	100% cases 3 cases <100 ng/mL
PRETEXT staging frequency	I (30%), II (30%), III (10%), and IV (30%)	I (5%), II (10%), III (45%), and IV (40%)
SIOPEL risk stratification	Standard risk = 6 High-risk = 4	Standard risk = 10 High-risk = 10
Histology types	Epithelial = 80% Mixed epithelial and mesenchymal = 20%	Epithelial = 55% Mixed epithelial and mesenchymal = 45%
Follow-up	Death = 10% Planned for transplant = 10% Lost to follow-up = 40% Doing well = 40%	Death = 35% Doing well = 35% Lost to follow-up = 25% Recurrence = 5%

*Note.* AFP = α-fetoprotein; SIOPEL = International Childhood Liver Tumors Strategy Group.

## Discussion

Primary malignant tumors of the liver account for roughly 1% of all childhood malignancies. Out of these, the most common type is hepatoblastoma, which has an annual incidence of 0.9 per 1 million children (21). 

The majority of hepatoblastoma cases are sporadic with no ostensible primary etiology. Syndromic associations are seen in 5% of cases, such as familial adenomatous polyposis, Beckwith-Wiedemann syndrome, and trisomy 18 (Edwards' syndrome). There is a convincing relationship with low birth weight and is highest when birth weight is <1000 g. Parenteral smoking is a major risk factor for low birth weight hence it becomes a risk factor for hepatoblastoma (22).

Hereditary aberrations of the WNT/beta-catenin signaling pathway are existent in around 80% of hepatoblastomas. These deviations include long deletions in exon 3 of Beta-catenin as well as alterations in CTNNB1, AXIN gene, and APC. Cyclin D1, survivin, and MYC, which are the focuses of WNT signaling, are overexpressed (23,24). One of the 2 subclasses of hepatoblastoma on gene expression profiling is allied with greater genetic instability (gains of chromosomes 8q and 2p), overexpression of hepatic progenitor cell markers (AFP, Cytokeratin 19, and EpCAM), and upregulated MYC signaling. These cancers parallel a more destructive clinicopathological phenotype with an advanced stage, superior proclivity for invasion and metastasis, and more poorly differentiated histological patterns. This cluster corresponds to immature histological phenotypes, such as embryonal and crowded fetal hepatoblastomas. The other assembly (which droughts the above-mentioned genetic modifications) chiefly entails the pure fetal phenotype (22).

Hepatoblastomas are typically self-contained lesions that subjugate 1 or more lobes of the liver or may transgress more than 1 liver segment (the basis for PRETEXT staging). PRETEXT assignment to 1 of 4 PRETEXT groups (PRETEXT I, II, III, or IV) is resolute by the number of adjoining uninvolved sections of the liver. PRETEXT is further interpreted with a V, P, E, M, or C letter, contingent upon the extension of the tumor beyond the hepatic parenchyma of the major sections. Caudate involvement is annotated as C. Tumor extension outside the liver to a contiguous intraabdominal organ (e.g., stomach and diaphragm) is annotated as E. Distant metastatic disease (usually lungs) is annotated as M. Major vascular involvement is annotated as V (all 3 hepatic veins or the vena cava) or P (portal bifurcation or the main portal vein). This classification is espoused by all International Liver Tumor Study groups (25,26).

On gross examination, hepatoblastomas appear as well-delineated single or multiple nodules with a nodular and bosselated appearance. On the cut surface, the consistency and hue depend on the tumor constituents and the presence or absence of necrosis and hemorrhage. Fetal hepatoblastomas display a tan-brown color that looks analogous to that of normal liver. In the other histological outlines, the cut surface is often variegated, with soft or gelatinous brown to red areas. When osteoid is present, the tumor is classically gritty, and the cut surface displays multiple white and slightly transparent speckles (22) (Figure 2).

International Pediatric Liver Tumors Consensus Classification has categorized hepatoblastoma into 2 main histological types: epithelial and mixed epithelial/mesenchymal types. The epithelial type is additionally categorized into several subtypes, such as fetal, embryonal, small cell undifferentiated (SCUD), cholangioblastic, and macrotrabecular patterns, occurring alone or in variable combinations (27). 

The fetal pattern contains thin trabeculae or lobules of small to medium-sized polygonal cells that resemble hepatocytes of developing fetal liver. The cytoplasm is clear or finely granular and eosinophilic, containing variable amounts of glycogen and lipids, consequently revealing a characteristic light and dark array at low magnification. The nucleus is small and round with fine nuclear chromatin and inconspicuous nucleolus. There are foci of extramedullary hematopoiesis poised of clusters of erythroid precursors. The fetal type typically has low mitotic activity. There is a subclass of fetal hepatoblastomas that show substantial mitotic activity. These reveal larger and more pleomorphic nuclei and dwindled cytoplasmic glycogen stemming from a crowded and hypercellular appearance. These are called mitotically active fetal hepatoblastomas (Figure 4).

The embryonal pattern resembles the developing liver at 6-8 weeks of gestation. The organization of the cells in this subtype is in the form of solid nests, lobules, or glandular/acinar structures, along with pseudo rosettes and papillary patterns. The cells demonstrate scant and dark granular cytoplasm with enlarged nuclei and coarse chromatin. Mitosis is more brisk in the embryonal type than in the fetal type. 

The SCUD pattern exhibits solid sheets of discohesive cells and shows abundant apoptosis, necrosis, and mitotic figures. This subtype can be both positive and negative for SMARCB1 (INI1). SMARCB1-positive SCUD tumors have a superior prognosis. SMARCB1-negative tumors undoubtedly characterize hepatic rhabdoid tumors and unveil features similar to those of rhabdoid tumors, such as higher stage at diagnosis, chromosomal deletions or translocations of 22q11, and low serum AFP levels.

The macrotrabecular pattern comprises thick trabeculae, 5-12 cells thick, and resembles the trabecular architecture of hepatocellular carcinoma. The trabeculae may be constituted of fetal or embryonal hepatoblasts or pleomorphic cells. In the cholangioblastic pattern, small ducts are lying within or around the hepatocellular components (Figure 6).

The mesenchymal pattern frequently is comprised of mature and immature fibrous tissue, osteoid or osteoid-like tissue, and hyaline cartilage. Limited cases exhibit teratoid features, comprising endodermal, neuroectodermal, incorporating, and melanin-producing cells, glial elements, neuronal cells, or complex mesenchymal tissues (22) (Figure 5).

Immunohistochemistry may benefit in risk stratification and guide treatment, as well as in ascertaining residuary neoplastic tissue and defining its boundaries in surgical specimens following chemotherapy. However, there is no marker to differentiate hepatoblastoma from hepatocellular carcinoma. Epithelial fetal and mesenchymal components may express nuclear and cytoplasmic beta-catenin due to stimulation of the WNT/beta-catenin signaling pathway. Less differentiated epithelial constituents may express AFP. Fetal components may also express Hep Par-1, which is negative in more immature epithelial components. Both fetal and embryonal components express glypican-3. Epithelial components can variably express pancytokeratins. SMARCB1-negative SCUD tumors have a worse prognosis (22).

One of the differentials for SCUD hepatoblastoma is the rhabdoid tumor, which entails the expanses of epithelioid cells with an eccentric nucleus and prominent periodic acid schiff stain-positive intracytoplasmic inclusions. These tumors show loss of SMARCB1 (INI1) expression, which relates to the deletion or deactivation of SMARCB1 on chromosome 22. Fetal hepatoblastoma must be differentiated from well-differentiated hepatocellular carcinoma, showing the presence of thickened trabeculae, higher N:C ratio, and absence of light and dark areas (22).

The SIOPEL risk stratification protocol stratifies all patients into standard risk and high-risk based on the following features: 

Standard risk: PRETEXT I, II, and III, those without metastasis, vascular invasion, or extrahepatic disease.

High-risk: PRETEXT IV, AFP <100 ng/mL, with extrahepatic disease or tumor rupture (28,29).

It is to be noted that the histopathological pattern is not included in the criteria for risk stratification. Our study indicated that the mixed epithelial and mesenchymal pattern was associated with poor prognosis and early death. Hence, we propose that the histopathological pattern could be considered as one of the criteria for risk stratification of patients, in addition to radiological and biochemical criteria in the SIOPEL system.

Most of the features were similar between our study and one of the largest studies on hepatoblastoma conducted by Hsu *et al.* (30) at the National Taiwan University Hospital (Table 2).

Compared to an Indian study by Archana *et al.* (31), the age at presentation, male-to-female ratio, and AFP levels were found to be in line with our study (Table 3).

The treatment algorithm followed internationally and at our institute is the SUPER PLADO regimen which consists of cisplatin, carboplatin, and doxorubicin infusion (Figure 1).

## Conclusion

PRETEXT staging and SIOPEL risk stratification play a major role in classifying patients into standard and high-risk. Based on our data, the histopathological pattern also correlated with the follow-up; hence, it can be considered one of the parameters in the risk stratification protocol. Due to the high mortality observed in high-risk cases, there is scope for more research to improve survival.

## Conflict of Interest

The authors declared no conflict of interest.
